# Impact of different manufacturers and gauge sizes on the performance of backflush needle

**DOI:** 10.1038/s41598-020-78668-6

**Published:** 2020-12-08

**Authors:** Hisanori Imai, Akira Tetsumoto, Hiroko Yamada, Makoto Nakamura

**Affiliations:** Division of Ophthalmology, Department of Surgery-Related, Kobe University Graduate School of Medicine, 7-5-2 Kusunoki-cho, Chuo-ku, Kobe 650-0017 Japan

**Keywords:** Eye diseases, Medical research

## Abstract

The present study aimed to identify the factors regulating aspiration rate (AR) of backflush needles and to compare those factors across various backflush needles from different manufacturers. The 27-gauge (27G), 25-gauge, and 23-gauge backflush needles from four different manufacturers, Alcon, MedOne, VitreQ, and DORC, were used for this experiment. AR was measured at four different aspiration vacuum levels: 100, 200, 400, and 650 mmHg. AR was significantly increased as the aspiration vacuum level increased when both aspirating balanced salt solution (BSS) and ethylene glycol; however, 27G products from VitreQ and MedOne were unable to aspirate ethylene glycol at the low aspiration vacuum level of 100 mmHg. When aspirating BSS at the high aspiration vacuum level of 650 mmHg, a smaller gauge number generally resulted in a significantly higher AR. AR, inner diameter, and cross-sectional area in Alcon products were significantly larger in any gauge, and the shaft length of Alcon products was significantly shorter than those of other manufacturers’ products in any gauge. Cross-sectional area negatively correlated with the degree of shaft deflection (*r*^*2*^ = 0.21, *p* = 0.042). These results imply that AR differs significantly among backflush needles and among companies depending on the shaft cross-sectional area.

## Introduction

Modern vitrectomy, also known as minimally invasive vitreous surgery (MIVS), has made great stride with the development of smaller gauge needles and high-speed cutters, the introduction of the trocar cannula system, and the advances in intraocular illumination and wide-angle viewing systems. As a result, small self-sealing sutureless incisions, reduced rates of perioperative complications, and early social rehabilitation after surgery have been achieved^1,2^.

MIVS requires small gauge surgical instruments; however, the quality of the instruments should not be compromised in smaller gauges. For example, the vitreous cutter used in 27-gauge (27G) vitrectomy was expected to present problems like reduced surgical efficiency and shaft stiffness. However, the vitrectomy efficiency improved dramatically by changing the drive system of the vitrectomy machine^3,4^ and by developing a bi-blade vitreous cutter^5,6^. Although the debate on shaft stiffness is still ongoing, an increasing number of reports have found that these instruments pose no problem in surgical procedures^7–11^. There were concerns about inadequate light intensity with the light pipe, although the light source was changed from halogen to xenon and/or mercury vapor and chandelier lighting has also become commonplace^12,13^, thereby securing sufficient light intensity. There are virtually no problems now regarding brightness of the surgical field during surgery. Thus, many recent reports have found that 27G vitrectomy is sufficiently practical and useful in the treatment of various vitreoretinal diseases, which was initially regarded as difficult^7–11^.

However, regarding other surgery-related instruments, including the backflush needle, operation with a larger gauge is still considered advantageous. The backflush needle is a tool that performs aspiration of subretinal fluid and drainage out of the vitreous cavity during vitrectomy to restore retinal detachment and macular hole. Therefore, reducing the gauge size of these needles would reduce the fluid volume aspirated per time unit, which would decrease the surgical efficiency. In fact, when performing 27G vitrectomy, the aspiration rate (AR) of both passive and active suction by a 27G backflush needle was significantly inferior to the AR of a larger gauge needle. However, understanding the advantages and limitations of the different backflush needles and selecting the optimal one is complicated for surgeons because there have been no reports comparing AR across various types of backflush needles. To select the best instrument for a vitrectomy procedure, it is essential for the surgeon to have information about the unique properties of each product.

This study aimed to compare the performance of backflush needles across various gauges of backflush needles from different manufacturers and to identify the factors that regulate AR of the active suction.

## Results

**Figure 1:**
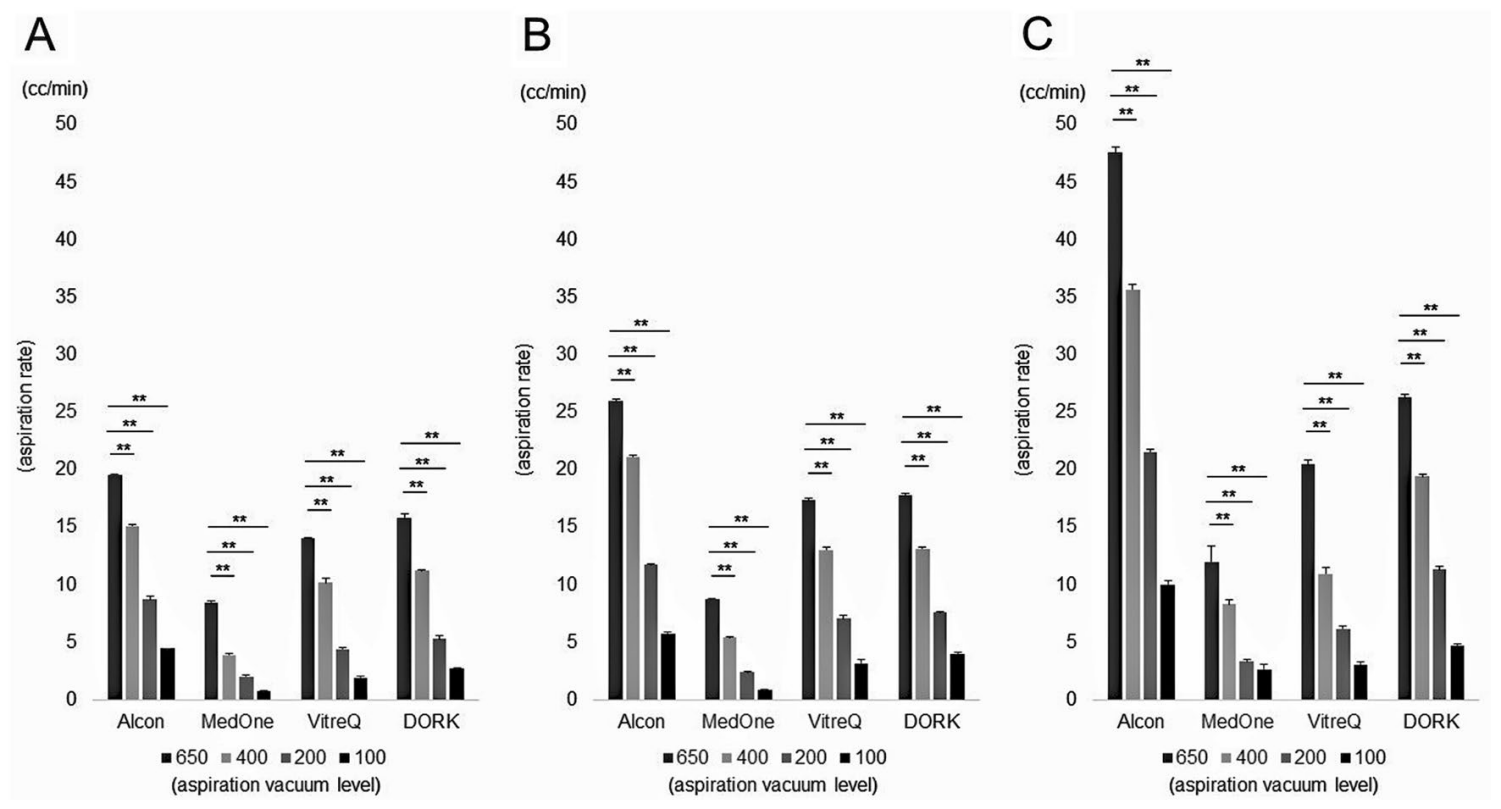
Comparison of aspiration rate (AR) under the condition of balanced salt solution aspiration. AR decreased significantly as the aspiration vacuum level decreased in each gauge of each company (**p < 0.01). (**A**: 27G, **B**: 25G, **C**: 23G).

**Figure 2:**
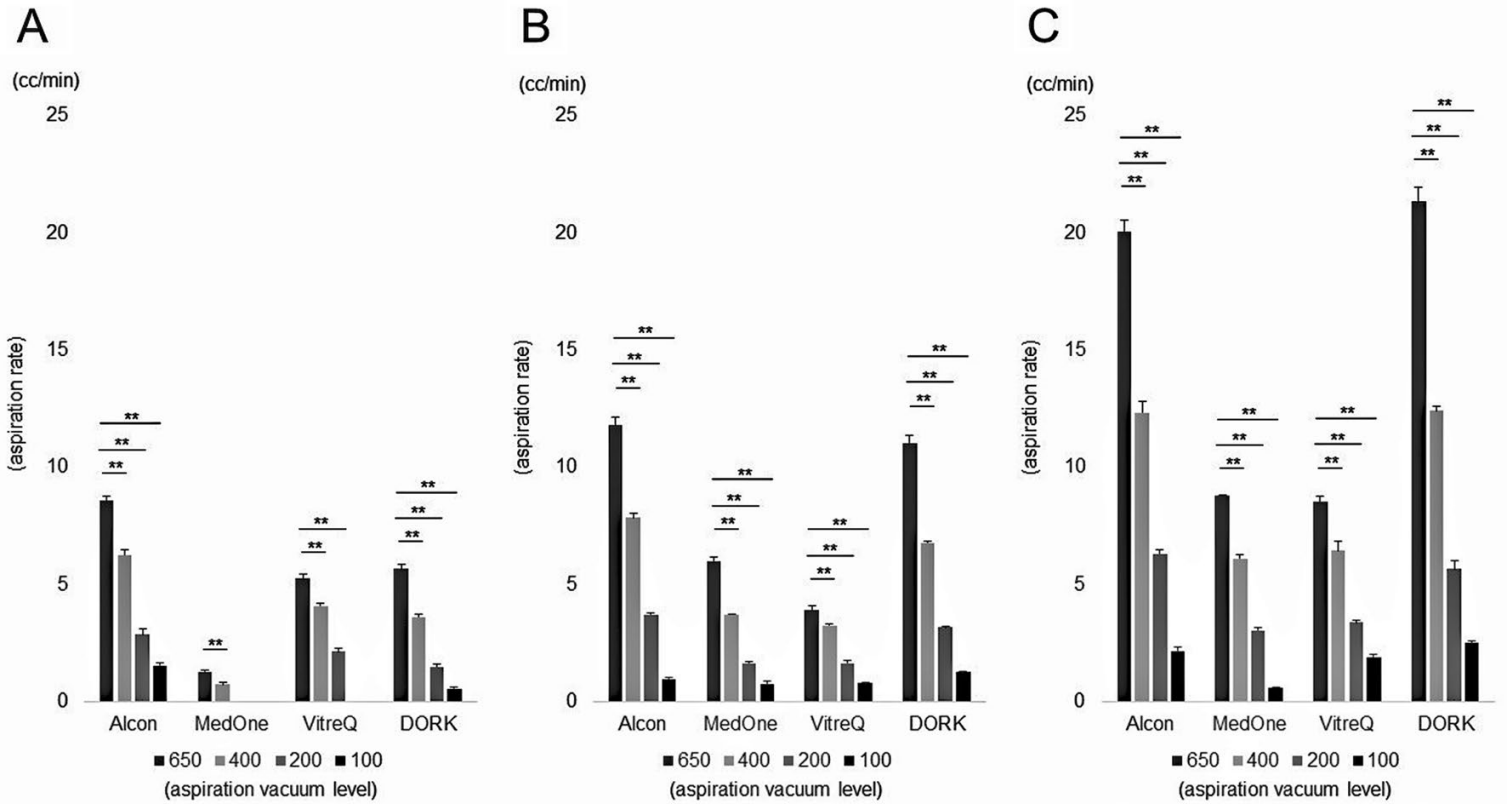
Comparison of aspiration rate (AR) under the condition of ethylene glycol aspiration. AR decreased significantly as the aspiration vacuum level decreased in 23G and 25G products in each company. In 27G product, AR decreased significantly as the aspiration vacuum level decreased in Companies A and D (**p < 0.01). 27G product from Company B (VitreQ) was unable to aspirate ethylene glycol at all at an aspiration vacuum level of 100 mmHg. In addition, 27G product from Company C (MedOne) could not aspirate ethylene glycol at all at the aspiration vacuum levels of 100 mmHg and 200 mmHg. (**A**: 27G, **B**: 25G, **C**: 23G).

First, we compared the AR for each aspiration vacuum level of each gauge from each company. As a result, when aspirating balanced salt solution (BSS), AR increased significantly as the aspiration vacuum level increased in each gauge from each company (Fig. 1). Similarly, when aspirating ethylene glycol, AR increased significantly at higher aspiration vacuum levels, with the exception of the 27G products from Companies B and C. The 27G product from Company B was unable to aspirate ethylene glycol at all at an aspiration vacuum level of 100 mmHg. Additionally, the 27G product from Company C could not aspirate ethylene glycol at all at the aspiration vacuum levels of 100 mmHg and 200 mmHg (Fig. 2). Overall, the aspiration rate when aspirating ethylene glycol, which is more viscous than BSS, was clearly lower than when aspirating BSS in each gauge of each company. From this result, we decided to use the BSS aspiration value at the aspiration vacuum level of 650 mmHg for the following analyses.

**Figure 3:**
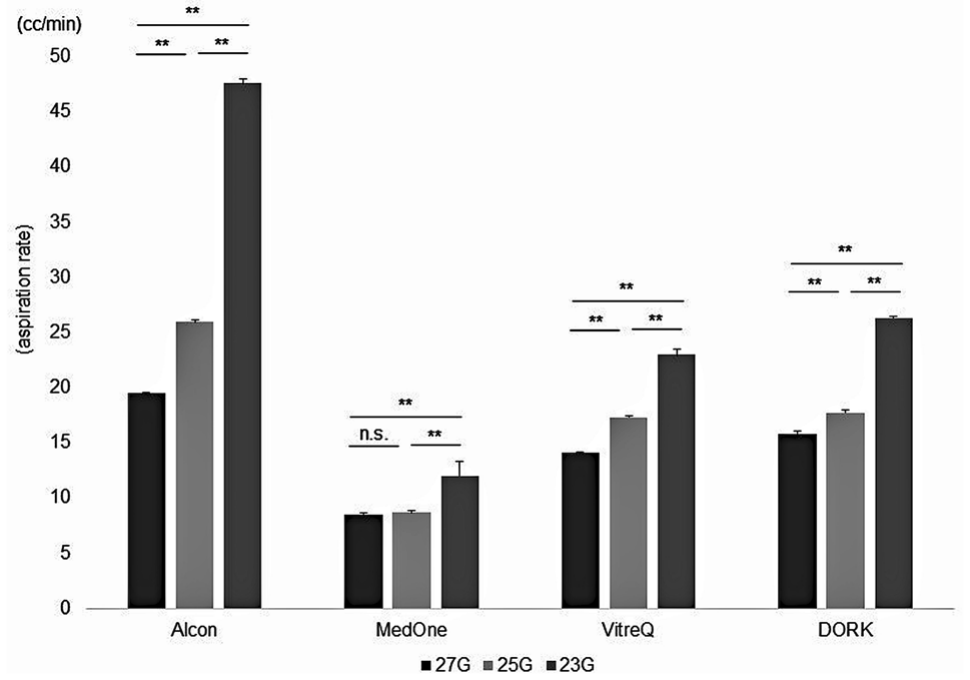
Comparison of aspiration rate (AR) between product gauges of each company. Except for the results of the comparison between the 27-gauge and 25-gauge products from Company B (MedOne), smaller gauge number resulted in significantly higher AR (**p < 0.01).

We compared AR between product gauges of each company. Except for the results of the comparison between the 27G and 25-gauge (25G) products from Company B, smaller gauge number resulted in significantly higher AR (Fig. 3). Next, we compared gauge size-dependent AR among manufacturers. Compared to other companies, AR in the products from Company A was significantly larger in any gauge (Table 1).

**Table 1:** Comparison of aspiration rate (cc/min) in each backflush needle.

The size of gauge	Company						
	Alcon	MedOne	*p* value	VitreQ	*p* value	DORK	*p* value
27	19.56 ± 0.04	8.46 ± 0.17	< 0.01	14.08 ± 0.04	< 0.01	15.85 ± 0.27	< 0.01
25	25.98 ± 0.12	8.71 ± 0.13	< 0.01	17.33 ± 0.15	< 0.01	17.73 ± 0.24	< 0.01
23	47.57 ± 0.47	12.01 ± 1.32	< 0.01	22.97 ± 0.56	< 0.01	26.35 ± 0.17	< 0.01

Table 2 shows basic shaft data of each product. Even within the same gauge size, each shaft had different characteristics from company to company. In particular, the internal diameter and the cross-sectional area of the products from Company A were significantly larger than those from other companies in any gauge. On the other hand, the shaft length of the products from Company A was significantly shorter than that from other companies in any gauge. The shaft deflection of the products from Companies B and C tended to be greater than that from Companies A and D regardless of gauge size. In 27G products, the shaft deflection of the product from Company A was significantly less than that of the products from Companies B and C, although there was no statistical difference with the product from Company D. In 25G products, the shaft deflection of the product from Company A was significantly less than that of other companies. In 23-gauge (23G) products, the shaft deflection of the product from Company D was significantly less than that of other companies.

**Table 2:** Basic shaft data of each backflush needle.

Size of gauge	Parameters of the shaft	Company						
		Alcon	MedOne	*p* value	VitreQ	*p* value	DORC	*p* value
27	Internal diameter (mm)	0.23 ± 0.00	0.16 ± 0.00	< 0.01	0.20 ± 0.01	< 0.01	0.16 ± 0.00	< 0.01
	External diameter (mm)	0.30 ± 0	0.30 ± 0.00	n.s	0.30 ± 0.00	n.s	0.30 ± 0.00	n.s
	Cross-sectional area (mm^2^)	0.05 ± 0.00	0.03 ± 0.00	< 0.01	0.04 ± 0.00	< 0.01	0.02 ± 0.00	< 0.01
	Length without tip (mm)	32.14 ± 0.09	40.50 ± 0.00	< 0.01	39.02 ± 0.04	< 0.01	37.98 ± 0.08	< 0.01
	Shaft displacement (mm)	8.9 ± 0.7	4.9 ± 0.2	< 0.01	5.2 ± 0.6	< 0.01	8.7 ± 0.4	n.s
25	Internal diameter (mm)	0.35 ± 0.00	0.31 ± 0.01	< 0.01	0.33 ± 0.00	< 0.01	0.34 ± 0.01	< 0.01
	External diameter (mm)	0.38 ± 0.04	0.40 ± 0.00	n.s	0.40 ± 0.00	n.s	0.38 ± 0.04	n.s
	Cross-sectional area (mm^2^)	0.12 ± 0.00	0.10 ± 0.00	< 0.01	0.11 ± 0.00	< 0.01	0.12 ± 0.01	< 0.01
	Length without tip (mm)	35.60 ± 0.00	38.72 ± 0.04	< 0.01	39.22 ± 0.54	< 0.01	40.88 ± 0.04	< 0.01
	Shaft displacement (mm)	7.8 ± 0.6	3.7 ± 0.8	< 0.01	3.3 ± 0.3	< 0.01	7.0 ± 0.6	< 0.05
23	Internal diameter (mm)	0.45 ± 0.01	0.39 ± 0.00	< 0.01	0.42 ± 0.01	< 0.01	0.43 ± 0.00	< 0.01
	External diameter (mm)	0.50 ± 0.00	0.50 ± 0.00	n.s	0.50 ± 0.00	n.s	0.50 ± 0.00	n.s
	Cross-sectional area (mm^2^)	0.20 ± 0.01	0.15 ± 0.00	< 0.01	0.17 ± 0.01	< 0.01	0.18 ± 0.00	< 0.01
	Length without tip (mm)	35.48 ± 0.11	39.02 ± 0.08	< 0.01	39.2 ± 0.17	< 0.01	41.20 ± 0.07	< 0.01
	Shaft displacement (mm)	3.1 ± 0.2	1.8 ± 0.4	< 0.01	2.5 ± 0.4	< 0.01	3.5 ± 0.4	< 0.05

**Figure 4:**
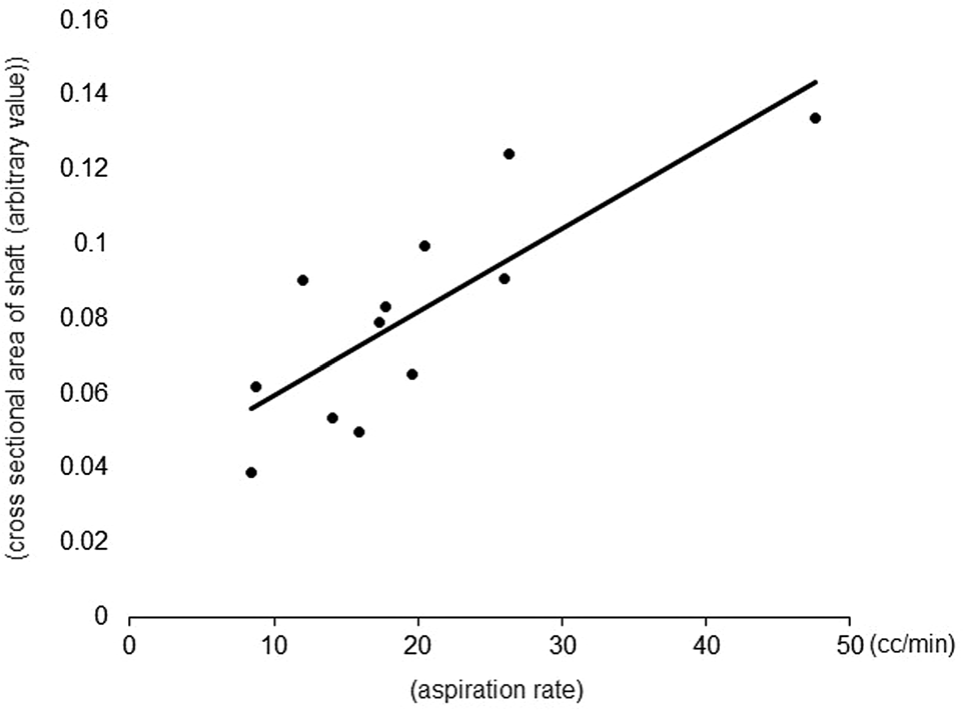
Correlation between aspiration rate (AR) and cross-sectional area of the shaft. AR positively correlated with cross-sectional area of the shaft (*r*^2^ = 0.75, *p* = 0.0002).

**Figure 5:**
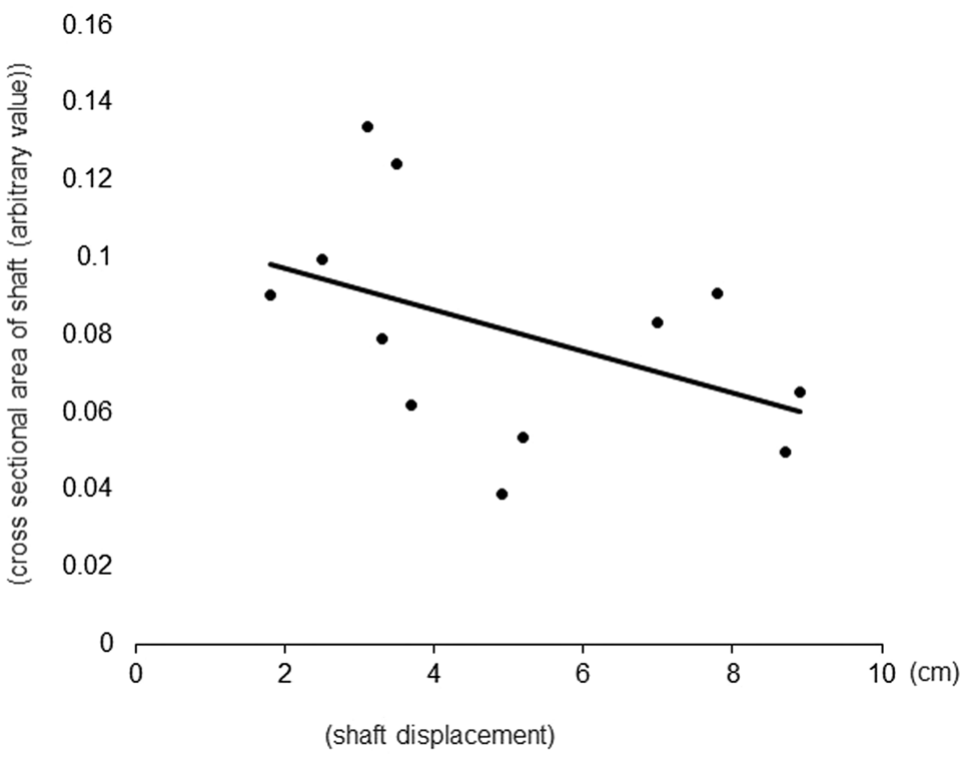
Correlation between shaft deflection and cross-sectional area of the shaft. Cross-sectional area of the shaft negatively correlated with the degree of shaft displacement (*r*^2^ = 0.21, *p* = 0.042).

AR positively correlated with cross-sectional area (*r*^2^ = 0.75, *p* = 0.0002; Fig. 4). Cross-sectional area of the shaft negatively correlated with shaft displacement (*r*^2^ = 0.21, *p* = 0.042; Fig. 5).

**Figure 6:**
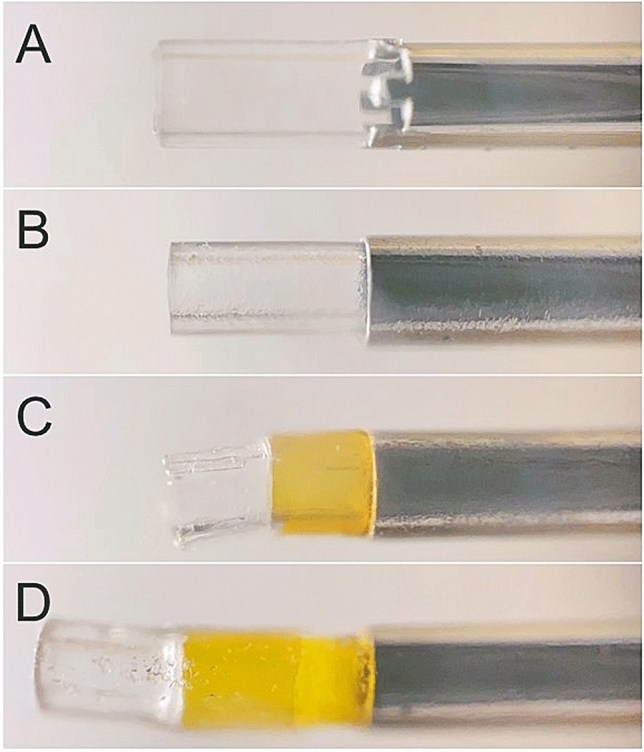
Magnified photographs of the joint between the silicon chip and the shaft in the 27G product from each manufacturer. The jointing method varied from company to company. The product from Company A (Alcon) had a structure in which a similarly processed silicon chip was fitted into the uneven metal part and the jointing surface was bonded with an adhesive (**a**). The products from Company C (VitreQ) (**c**) and Company D (DORK) (**d**) had a structure in which the shaft and the silicon chip were bridged and jointed by a tube of resin material. Detailed information was not available for the product from Company B (MedOne) (**b**).

In 27G products, the method of jointing the silicon tip to the shaft varied from company to company. The product from Company A had a structure in which a similarly processed silicon chip was fitted into the uneven metal part and the jointing surface was bonded with an adhesive. The products from Companies C and D had a structure in which the shaft and the silicon chip were bridged and jointed by a tube of resin material. Detailed information was not available for the product from Company B (Fig. 6).

The shafts of Companies A, B and D are made of stainless steel, and the shafts of Company C are made of alloy steel comprising nickel, cobalt, chromium and molybdenum. Neither shaft has been machined or coated on the inner or outer surface. Detailed information about Young's modulus was not available for any of the products from any of the companies.

## Discussion

Technological advances in the field of vitrectomy, represented by the evolution of MIVS, imply that surgeons have more options regarding surgery settings. At the same time, the goal of any vitrectomy is to perform the desired intraoperative intervention while minimizing collateral damage in the most efficient way possible. Therefore, it is important to understand the principles of the technology, including the characteristics of backflush needles.

First, AR was measured for each aspiration vacuum level of each manufacturer under both BSS aspiration and ethylene glycol aspiration conditions; therefore, AR relied on aspiration vacuum levels in both BSS and ethylene glycol aspiration conditions. Additionally, AR when aspirating ethylene glycol was obviously lower than AR when aspirating BSS. Particularly, the 27G products of Companies B and C were unable to aspirate ethylene glycol at all at low aspiration vacuum level. Clinically, during surgery for the treatment of retinal detachment, especially for old retinal detachment, it is frequently necessary to aspirate a highly viscous liquid vitreous using the backflush needle to reattach the retina. Our data may suggest that aspiration at low aspiration vacuum levels using a smaller gauge backflush needle could result in incomplete drainage of viscous subretinal vitreous liquid and retina re-detachment after surgery. We also investigated the AR of different backflush needles from different manufacturers. In the study of vitreous cutters, it is reported that AR greatly contributes to surgical efficiency^6,14–17^. Especially if the AR is low, an important advantage of MIVS, the shortening of the operation time, may be affected, which may lead to an extension of the operation time. Therefore, it is important to examine the difference in AR between products even in backflush needles. Our study found that when comparing backflush needles manufactured by the same company, AR increased significantly as the gauge size increased. This result was in line with the expectations, as it has been reported in various studies of vitreous cutters that gauge size is one of the critical features determining actual flow rate of vitreous cutters^17^. On the other hand, even if the gauge size is the same, the AR varies significantly from company to company. It has also been reported that for 20G-, 23G-, and 25G cutters, AR and internal diameter vary from company to company even for cutters of the same gauge^17^. Therefore, we measured the internal diameter and cross-sectional area of the shaft of each company's products. The results indicated that, even for the same gauge size, the internal diameter and cross-sectional area of the shaft differed significantly from company to company. The AR was significantly correlated with the cross-sectional area. This result suggests that even within the same gauge sizes, the thickness of the metal varies from company to company, and this may contribute to the difference in AR. Therefore, the results of this study are important reference data for determining the type of backflush needle that should be selected for surgery.

Next, we compared the shaft deflection, which is a contributing factor to the shaft stiffness, across various backflush needles from different manufacturers. Generally, the reduction of the instrument caliber resulted in increased flexibility^17,18^. It is possible that a less rigid instrument causes a difficult movement control, particularly in the case of the 27G backflush needle. Therefore, stiffness is a crucial parameter of all intraocular instruments, including the backflush needle, for the performance of accurate surgical maneuvers. In this study, our results have demonstrated that shaft deflection was negatively correlated with shaft cross-sectional area. This result suggests that for the same gauge size, the larger the shaft cross-sectional area, the greater the AR, as mentioned above, while the shaft stiffness is conversely compromised. Also, the shaft length of the product from Company A was significantly shorter than that of the other companies in any gauge. In eyes with a normal axial length, both the shaft stiffness and shaft length may not affect surgical performance, although in eyes with long axial length over 30 mm, careful consideration should be taken for product selection.

Finally, we examined the silicon chip bonding method, which determines the easiness to remove from and insert into the cannula and the tip durability during these procedures. The results indicated that the silicon chip bonding method, especially for 27G products, seems to vary among companies. The impact of this different joining method has not been investigated in detail, but it may be related to the fragility due to which the silicon tip may be unexpectedly detached from the shaft during surgery. Further detailed examination is required in the future. Hence, considering these points, although the data on AR obtained in this study may serve as important reference data, it is necessary for each surgeon to decide on the product after carefully examining each of them.

The study has the following limitations: First, errors may have occurred because AR and the length of each part of the shaft were only measured once, manually, per each product. Second, detailed data on how silicon tips are bonded was not available for one company; therefore, its durability could not be fully verified. Third, the shaft stiffness was not calculated because we were unable to procure have enough information regarding the material of the shaft, especially the Young’s module of the materials. We believe this to be a topic for future studies. However, we believe our results about shaft deflection still have enough information to discuss the stiffness of the shaft. Finally, the data are based on basic experiments and may differ from the actual clinical situation. However, despite the fact that many products have been launched so far, important factors such as AR and shaft stiffness have not been published as far as we know, and we believe that the data obtained in this study are very informative.

In conclusion, we compared the performance of backflush needle in different gauges and different manufacturers. It was found that AR differed significantly among backflush needles and among companies depending on the shaft cross-sectional area. This study is considered to be important reference data when deciding which product to use for surgical procedures.

## Methods

Because this is nonclinical research, approval by an Ethics Committee was not required. The following 27G, 25G, and 23G backflush needles were used for this study: Company A, Advanced DSP Backflush Soft Tip (Alcon Grieshaber AG, Schaffhausen, Switzerland), Company B, Backflush FlexTip (MedOne Surgical, Inc., Sarasota, USA), Company C, Brush Backflush Instrument (VitreQ BV, Vierpolders, The Netherlands), and Company D, Backflush instrument (DORC. International, Zuidland, The Netherlands).

The following parameters were measured for analysis: external diameter, internal diameter, full length, cross-sectional area, and shaft deflection. To measure the external diameter, the internal diameter, and the full length of the shaft, we first disassembled the shafts of each product. First, the length of disassembled shafts was measured using a digital caliper (Shinwa digital NOGISU; Shinwa Rules Co., Ltd., Niigata, Japan) with an accuracy of 0.1 mm, and this was defined as “full length of the shaft.” Next, the disassembled shafts were positioned vertically under a smartphone camera equipped with a microscope (Nurugo Micro Smartphone Microscope; Nurugo), which took images at 400 × magnification. Both the external and internal diameters were calculated by using an image analysis software (ImageJ 1.51j8; National Institutes of Health, Bethesda, MD, USA). Afterwards, the external diameter measurement of each shaft was confirmed using the digital caliper (Shinwa digital NOGISU; Shinwa Rules Co., Ltd., Niigata, Japan). The squared internal diameter was calculated and used as the value to represent the cross-sectional area. We prepared three backflush needles for each gauge from all four companies, measured all parameters, and used it for analysis.

The shaft deflection, which can be a measure of the overall bendability of the metals that make up the shaft and is one of the contributing factors to the shaft stiffness, of each product was evaluated by measuring the tip displacement under a known force (0.5 N) at 20 mm from the shaft base as described previously^14^. The degree of deflection is proportional to the added force and the cube of the distance from the base to the point of the force. On the other hand, the degree of deflection is inversely proportional to Young's modulus and the moment of inertia of area. In this study, we kept the force and the distance from the base to the point of the force constant as described above. Thus, our results indicate that the greater the deflection, the more likely the material is to bend because both Young's modulus and the moment of inertia of area are numerical values related to the material and shape of the shaft material. The shaft displacement was measured using a digital caliper (Shinwa digital NOGISU; Shinwa Rules Co., Ltd., Niigata, Japan). We prepared five backflush needles of all gauges from all four companies, measured the shaft deflection, and used it for analysis.

AR was measured at aspiration vacuum levels of 100 mmHg, 200 mmHg, 400 mmHg, and 650 mmHg on all products. Usually, during surgery, the backflush needle is used for the aspiration of BSS in vitreous cavity and/or subretinal viscous liquid vitreous. The viscosity of BSS is about 1 centipoise (cps). On the other hand, the mean maximum viscosity of liquid vitreous in the subretinal space of retinal detachment was reported as 29.3 cps at 37° C^19^. Therefore, we used ethylene glycol, the viscosity of which was reported as 31.5 cps at 10° C, as an alternative material of subretinal viscous liquid vitreous for this study. Briefly, the 27G, 25G, and 23G products from each manufacturer were prepared. The beaker was put on an electronic scale, and each product was hung and set so as not to give any weight report on the electronic scale (i2000; ID IDAODAN). BSS or ethylene glycol in a beaker was aspirated for 30 s by each product using the Constellation Vision System (Alcon Laboratories, Inc., Fort Worth, TX, USA). BSS and ethylene glycol were aspirated at normal room temperature and 10° C, respectively. The amount of reduced liquid was measured using the electronic scale (i2000; ID IDAODAN), and twice that amount was used as AR (g/min). We prepared three backflush needles of all gauges from all four companies, measured ARs, and used it for analysis.

For the 27G product, by enlarging and photographing the joint with a smartphone camera equipped with a microscope (Nurugo Micro Smartphone Microscope; Nurugo), the difference in the method of jointing the silicon chip and the shaft at each company was visually confirmed.

Finally, we investigated the composition of the metal used for the shaft, the presence or absence of processing and/or coating on the outer and inner surfaces of the shaft.

### Statistical methods

Kruskal–Wallis H-test, followed by a post hoc analysis using Mann–Whitney U test with Bonferroni correction was used to compare AR and all parameters of the shaft. Spearman correlation analysis was then used to identify a significant correlation between shaft deflection and shaft cross-sectional area, and AR and cross-sectional area. Statistical analyses were performed using SPSS software (version 24.0, SPSS Inc., Chicago, IL). Statistical significance was inferred for *p* < 0.05.

## Data Availability

The datasets generated during and/or analyzed during the current study are available from H. Imai, the corresponding author on reasonable request.
